# Formamidinium Halide Perovskite and Carbon Nitride Thin Films Enhance Photoreactivity under Visible Light Excitation

**DOI:** 10.1021/acs.jpca.2c02565

**Published:** 2022-06-02

**Authors:** Gopi Ragupathy, Julian Rieß, Bat-El Cohen, Lioz Etgar, Roey Sagi, Kumar P. Deepak, Reinhard Schomäcker, Micha Asscher

**Affiliations:** † Institute of Chemistry, Edmund J. Safra Campus, Givat-Ram, The Hebrew University of Jerusalem, Jerusalem 91904 Israel; ‡ Department of Multiphase Reaction Technology, Technical Chemistry, Institute for Chemistry of the TU, Berlin, 10623 Germany

## Abstract

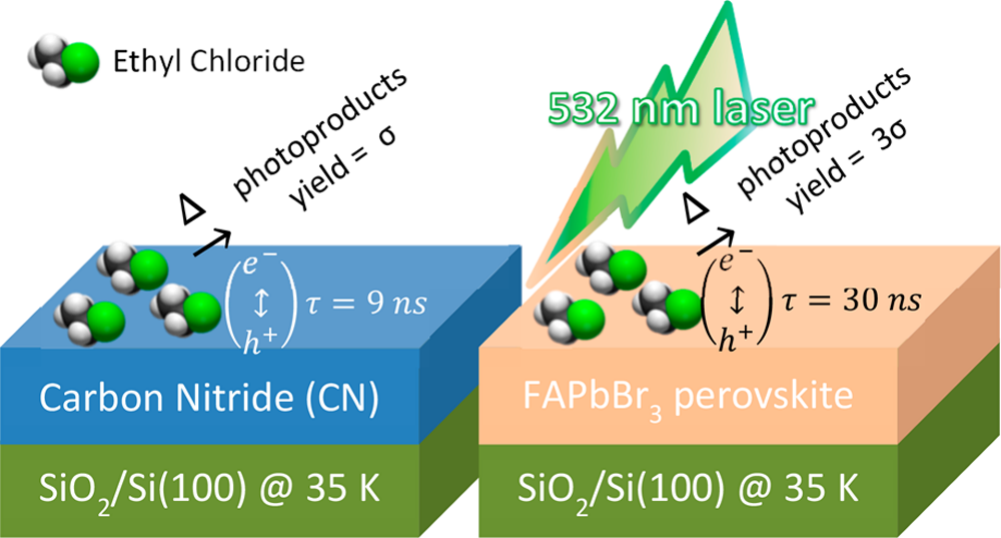

Photochemical and photocatalytic activity of adsorbates on surfaces is strongly dependent on the nature of a given substrate and its resonant absorption of the (visible) light excitation. An observation is reported here of the visible light photochemical response of formamidinium lead bromide (FAPbBr_3_) halide perovskite and carbon nitride (CN) thin-film materials (deposited on a SiO_2_/Si(100) substrate), both of which are known for their photovoltaic and photocatalytic properties. The goal of this study was to investigate the role of the substrate in the photochemical reactivity of an identical probe molecule, ethyl chloride (EC), when excited by pulsed 532 nm laser under ultrahigh vacuum (UHV) conditions. Postirradiation temperature-programmed desorption (TPD) measurements have indicated that the C–Cl bond dissociates following the visible light excitation to form surface-bound fragments that react upon surface heating to form primarily ethane and butane. Temperature-dependent photoluminescence (PL) spectra of the FAPbBr_3_ films were recorded and decay lifetimes were measured, revealing a correlation between length of PL decay and the photoreactivity yield. We conclude that the FAPbBr_3_ material with its absorption spectrum in resonance with visible light excitation (532 nm) and longer PL lifetime leads to three times faster (larger cross-section) photoproduct formation compared with that on the CN substrate. These results contrast the behavior under ambient conditions where the CN materials are photochemically superior due, primarily, to their stability within humid environments.

## 1 Introduction

Photocatalytic materials enable us to address several global environmental problems, such as the degradation of organic pollutants and wastewater purification.^1−14^ A need to develop easy-to-produce, cost-effective, and environmentally tolerant photocatalysts remains a major challenge. Among the potential photovoltaic and photoactive materials, halide perovskites^4−9^ and carbon nitrides^10−14^ are attractive new ones.

Simple fabrication methods, defect tolerance, tunable band gap, high absorption coefficient in the visible spectral range, and relatively long charge carrier lifetime keep halide perovskites at the forefront of photovoltaics research. Bromide-based perovskites have a relatively wide bandgap, emit green light, and are more stable than iodide-based counterparts, in particular for PV applications, specifically in more humid environments.^15,16^ An interesting example from the bromide-based perovskite family of materials is formamidinium lead bromide (FAPbBr_3_). This material exhibits bright photoluminescence with a peak at 550–560 nm and a relatively long exciton decay lifetime, up to 200 ns in a colloidal environment, and in the 10–30 ns range in supported films, apparently at a proximity to unique surface sites and long charge-carrier diffusion lengths. Although stated specifically for CH_3_NH_3_PbBr_3_ halide perovskite material, we believe that the same arguments hold for FAPbBr_3_ as well. The FAPbBr_3_-based films were demonstrated as efficient solar-cell materials.^15−17^ Based on such properties, the FAPbBr_3_ films are considered to be superior optoelectronic and PV materials, e.g., compared to the case of CH_3_NH_3_PbBr_3_.^1−4,16,17^

We have recently discovered a new attribute of halide perovskite materials, namely their photochemical activity under ultrahigh vacuum (UHV) conditions when activated by visible light. Using inorganic CsPbBr_3_ and organic CH_3_NH_3_PbBr_3_ halide perovskites, photodissociation of adsorbed ethyl chloride (EC) as a probe molecule was reported.^18^ As a reference, the photofragmentation of EC molecules adsorbed on Ag nanoparticles on top of a SiO_2_/Si(100) substrate has been studied in the same experimental setup using a visible pulsed laser excitation (532 nm) procedure, showing absolutely no activity in the visible light range.^19^ Photodissociation was observed only when UV wavelengths (355 nm or shorter) were employed.

Mesoporous polymeric carbon nitride (CN) has recently emerged as a promising photocatalyst (e.g., for water splitting) because of its simple and low-cost synthesis route, nonmetallic conjugated structure, nontoxic properties, porosity, high surface area, and high level of stability.^10−14^ It has a relatively narrow band gap of ∼2.7 eV, which endows it with an adequate light absorption capacity. Furthermore, the inherent functional groups, vacancies, and sp^2^-hybridized carbon network are essential for the generation and migration of delocalized electrons. Also, carbon nitride possesses a two-dimensional structure and six nitrogen lone-pair electrons which are favorable for the immobilization of metal species.^12−14^ On the basis of these excellent characteristics, carbon nitride photocatalysts have been applied in advanced oxidation processes, mostly to degrade organic pollutants from water.^20,21^ Furthermore, carbon nitride has been applied for the evolution of hydrogen from water splitting, as early studies have shown, as an element within solar cells, fuel cells, and energy storage.^22−24^ More recent studies^25,26^ have demonstrated that composite materials such as Co-MoS_2_ within C_3_N_4_ result in significant enhancement of hydrogen production via water splitting. Another application results in porous Mo material due to reactivity with the C_3_N_4_ template, also leading to enhanced hydrogen formation.^26^

Here we report on the photochemical activity of EC molecules (as a probe) adsorbed on FAPbBr_3_ halide perovskite films and compare them to similar films made of carbon nitride (CN), attempting to explore the role of the underlying substrate in the photoreactivity outcome with an emphasis on the effect of different exciton recombination lifetimes (directly measured and reported here) between the two substrates. Furthermore, this research allowed us to investigate the effect of visible light on these samples from a surface science perspective, enabling better understanding of the fundamental aspects of processes such as photochemistry and photoluminescence (PL) under well-defined conditions.

## 2 Experimental Section

### 2.1 Preparation of Formamidinium Lead Bromide (FAPbBr_3_) Films

#### 2.1.1 FABr Synthesis

Formamidinium bromide (FABr) was synthesized by reacting formamidine acetate salt (Aldrich) with excess hydrobromic acid (48 wt % in water, Aldrich) in a round-bottom flask at 50 °C for 1 h using continuous stirring, followed by solvent removal in a rotary evaporator. The precipitate was washed and cleaned five times with diethyl ether to obtain the clean FABr salt. More details on the exact synthesis procedure are provided in Supporting Information.

Mixing PbBr_2_ with the FABr salt led to the perovskite formation, where the final solution contained 1 M PbBr_2_ (Aldrich) and FABr (1:1) in DMF:DMSO solvents (85:15). These mixed solutions were used to form the halide perovskite FAPbBr_3_ film (see below).

#### 2.1.2 Halide Perovskite Film Deposition

FAPbBr_3_ perovskite film was prepared in a nitrogen-filled glovebox by using the solvent engineering technique reported elsewhere.^16^ A 50 μL amount of the filtered perovskite solution (employing a PTFE 45 μm filter) was spin-coated on top of a SiO_2_/Si (100) substrate that was transformed to porous silicon (PSi) via an electrochemical process in HF/ethanol solution. Typical pores were ∼20 nm wide and 2 μm deep. PSi has been employed to improve the adherence of the halide perovskite films to the silicon substrate. The perovskite film was deposited by using a dynamic spin program (5 s loading time followed by 10 s spin at 1000 rpm, which subsequently increases to 5000 rpm for 50 s). Thirty seconds before the program ended, anhydrous chlorobenzene (100 μL, Aldrich) was added dropwise on the substrate. For complete crystallization, the film was annealed for 30 min at 100 °C. The quality of the resulting films is shown in Supporting Information Figure S1A,C, where the XRD quality and the SEM image of the film are demonstrated.

### 2.2 Carbon Nitride

Mesoporous carbon nitride (CN) was prepared using published procedures.^12−14^ Cyanamide (CA) and tetraethoxysilicate (TEOS) were mainly used as sources to produce carbon nitrides. A certain amount of CA was dissolved in 0.01 N HCl (4 g) and ethanol (4 g) while the pH was adjusted to 2 with 1 N HCl solution. Afterward, the required amount of TEOS (1:6, TEOS:CA) was added and the mixture was stirred for 30 min. After evaporation of the solvents, the resulting film was heated to 80 °C for 24 h with subsequent heating to 550 °C in an argon atmosphere and heat-treated at this temperature for 4 h. Finally, CN_6_ was obtained by removing silica using NH_4_HF_2_ solution for 40 h and subsequent washing with water for several times and finally with ethanol.^12−14^

### 2.3 Carbon Nitride Film Deposition

Electrophoretic deposition (EPD) was carried out based on published procedures.^12−14^ Thirty mg of mesoporous carbon nitride, 30 mg of iodine, and 50 mL of acetone were added to a 100 mL beaker. The solution was then treated for 10 min in an ultrasonic bath. Subsequently, the beaker containing the solution was placed in the EPD unit. A 1 cm × 2 cm SiO_2_/Si(100) substrate, with a 1 cm^2^ circular porous silicon (PSi) area, etched at its center, was connected as the working electrode, while a second identical sample was attached as the counter electrode. Both electrodes were then connected to a potentiostat. One deposition cycle took 5 min by an applied potential of 10 V, and we performed three such runs per sample. The electrodes were subsequently removed from the solution, and the coated wafer was dried at 60 °C for 24 h in a vacuum oven. The resulting PSi sample contained a layer of 0.7 mg of carbon nitrite (about 5 μm thick).

### 2.4 UHV Photochemistry Experiments

The experimental setup for the photochemistry of ethyl chloride was described in a previous publication in detail.^18^ Briefly, an ultrahigh vacuum (UHV) chamber at a base pressure of ∼2× 10^–10^ Torr was the main environment for the photochemistry studies. This chamber is equipped with a quadrupole mass spectrometer (SRS, RGA-200) that is glass shrouded with a 5 mm orifice at its end, enabling temperature-programmed desorption (TPD) measurements at 1 mm distance from the sample, thus avoiding detection of desorbing molecules from nonsample surfaces. The high harmonics of a Nd:YAG laser provided photons at 532 nm (2nd), 355 nm (3rd), and 266 nm (4th) at a typical power of 1–2 mJ/cm^2^ per pulse (Spectra-Physics INDI 20).

The photodissociation of ethyl chloride (EC) was detected via postirradiation TPD measurements performed by tracing the desorption of the most probable photoproducts ethane (C_2_H_6_, *m*/*z* = 30), allyl radical (C_3_H_5_, *m*/*z* = 41), and propyl radical (C_3_H_7_, *m*/*z* = 43). Masses *m*/*z* = 41 and *m*/*z* = 43 are assigned to electron-impact fragments of butane (C_4_H_10_), formed within the quadrupole ionizer. It is important to note that no other products were detectable following exposure to the laser irradiation at any of the above wavelengths.

Following each of the photochemistry cycles, the surface was cleaned by gentle ion-sputtering at 300 K (Ne^+^ ions at 600 V and sample current of ∼2 μA for 10 min). From our previous studies, we concluded that no more than 15 such sputter cycles could be performed before damage to the sample becomes apparent.^18^

### 2.5 Luminescence Studies

Photoluminescence (PL) studies were performed following 355 nm pulsed laser irradiation (third harmonics of Nd:YAG laser at a power of 1 mJ/cm^2^ per pulse) as the light excitation source. The luminescence signals were detected by employing a fiber optic-based spectrometer (Ocean Optics USB 2000+) from outside the UHV chamber through the optical viewport at a spectral resolution of about 2 nm. PL spectra were obtained also from the Horiba instrument described below.

### 2.6 PL Lifetime Measurements

Following the preparation of both the FAPbBr_3_ and the carbon nitride (CN) films on top of the PSi substrate (for enhanced adhesion and stability of the films on the substrate), their PL lifetimes were measured ex situ, as follows: Measurements were conducted with a Horiba Scientific Fluoromax-4 spectro-fluorometer, and excitation was performed by NanoLED at 375 nm, having a resolution of 55 ps/channel. Emission was collected at 540 nm at a scale time of 800 ns, with time-correlated single-photon counting (TCSPC) detection.

## 3 Results and Discussion

### 3.1 Ethyl Chloride (EC) Adsorption on the Surfaces of FAPbBr_3_ and Carbon Nitride (CN)

**Figure 1:**
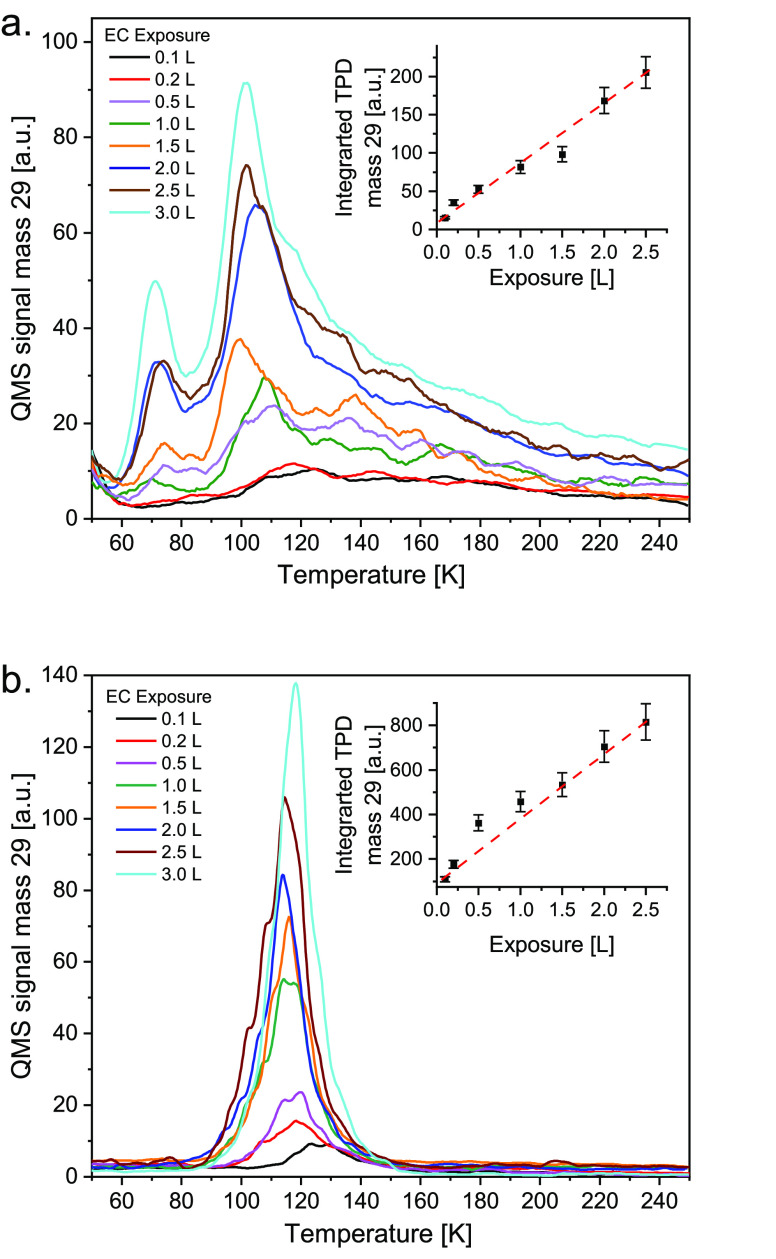
Temperature-programmed desorption (TPD) of ethyl chloride from (a) FA and (b) CN substrates (w/o irradiation): TPD spectra of EC at the indicated exposures in Langmuirs (L) (1 L = 10^–6^ Torr s). Exposure values were corrected for the ion gauge sensitivity factor. Heating rates were 1 K s^–1^. The inset in both a and b reflect the integrated area under the TPD peak at *m*/*z* = 29 vs exposure.

Previous research has shown that ethyl chloride molecules can adsorb on the surface of halide perovskites.^18^ TPD spectra obtained from different exposures of EC on the FAPbBr_3_ sample, kept at 35 K, are shown in Figure 1. The gas exposures are defined in Langmuir units (L), where 1L = 10^–6^ Torr s. In this paper, we compared the interaction of the EC molecules with the surfaces of FAPbBr_3_ and CN, with an attempt to explore the effect of the substrate on the visible light photoresponse with EC as a probe molecule. The role of exciton lifetime (measured via the photoluminescence lifetime) on the EC photodissociation yields is emphasized.

TPD spectra of the parent EC molecule (detected at mass 29, see Figure 1) from clean SiO_2_/Si (100) reveal a single desorption peak at 85 K (not shown). In the current samples, under identical conditions, two desorption peaks around 70 and 110 K are found from FAPbBr_3_ (Figure 1a) and a single TPD peak at 120 K on carbon nitride as the substrate (Figure 1b). We estimated that exposure of 1.5 Langmuirs (L) EC led to 1 ± 0.2 ML (monolayer) EC coverage on both the halide perovskite and the carbon nitride substrates, assuming a similar sticking probability of EC, approaching unity, on both substrates as indicated from the linear growth of the area under the TPD peak vs exposure at *m*/*z* = 29 (see insets in Figure 1a,b). Qualitative Redhead-like analysis reveals binding energy of 6.5 ± 1.0 kcal/mol and 7.1 ± 1.0 kcal/mol of EC to FAPbBr_3_ and the CN surfaces, respectively, at their monolayer coverage.

#### 3.2.1 Substrate Effect on the Photodecomposition of EC

**Figure 2:**
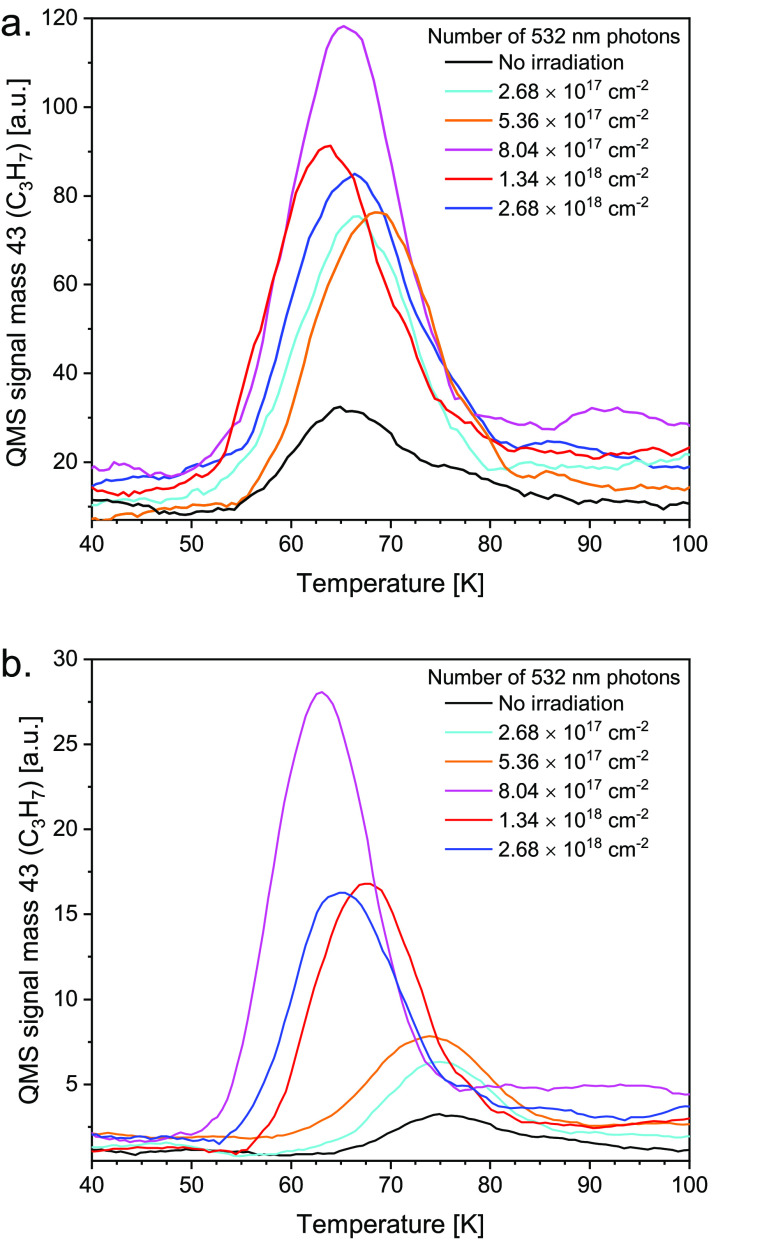
Postirradiation TPD spectra (1.5 L exposure of EC) of the photoproduct propyl radical (mass 43, C_3_H_7_) at the indicated number of 532 nm photons striking the (a) FAPbBr_3_ (b) CN substrates. The heating rate was 2 K/sec.

The photochemical behavior of halide perovskites and mesoporous carbon nitride substrates has been analyzed by tracking the photodissociation of EC molecules (and its photoproducts) by employing TPD measurements. These were performed following visible light (532 nm) irradiation as a function of the number of photons striking the FA or CN films. We recorded the parent molecule (1.5 ML of EC) and the most abundant photoproducts. Specifically, ethane (C_2_H_6_, *m*/*z* = 30), allyl radical (C_3_H_5_, *m*/*z* = 41), and propyl radical (C_3_H_7_, *m*/*z* = 43) were monitored; see Figure 2a,b.

These spectra indicate that the parent molecule population decreases as the number of pulses grows to 2.7 × 10^18^ photons, with a simultaneous increase in the population (coverage) of the photoproducts (shown in Figure 2 as *m*/*z* = 43). The propyl radical desorb as a peak at 55–80 K, and the ethane (*m*/*z* = 30) desorbs at 50 K (details in Supporting Information Figure S9), whereas the allyl radical (*m*/*z* = 41) desorbs at 80–100 K from FAPbBr_3_. Similarly, TPD peaks of the above products from the CN substrate are observed at 60–90 K (desorption of propyl), 50 K (desorption of ethane), and 70–100 K (desorption of allyl). As can be seen (details in Supporting Information Figure S9), propyl radicals exhibit similar TPD spectra to those of the allyl radicals, indicating that both of them originate from the same precursor parent molecule butane (C_4_H_10_, *m*/*z* = 58), and these two masses, therefore, are the result of fragmentation within the QMS.^18^

**Figure 3:**
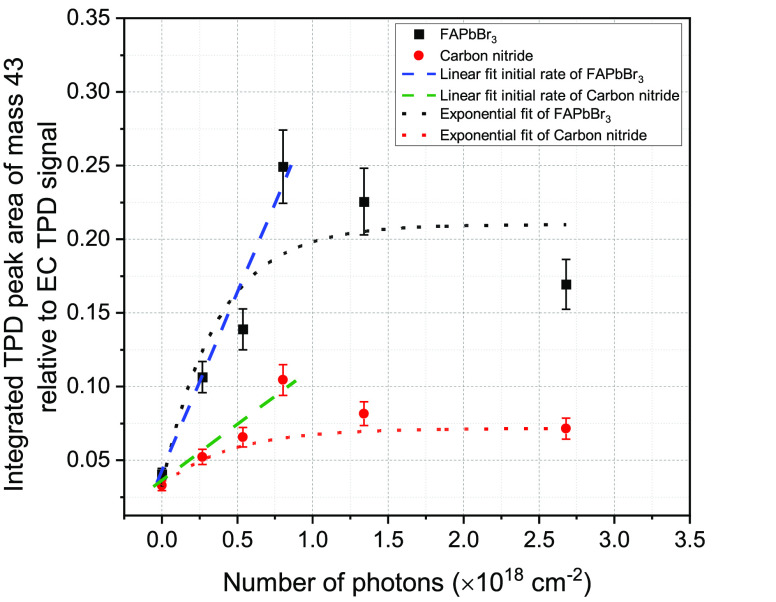
Integrated TPD peaks of mass 43 (propyl) vs the number of visible (532 nm) light photons striking the FAPbBr_3_ halide perovskite and carbon nitride (CN) substrates. The probe molecule is ethyl chloride (EC) in all cases.

In Figure 3, we compare the integrated area under the TPD spectra of these most abundant photoproducts (normalized to that of the parent EC molecule without irradiation). The yield of the photoproducts increases with the increasing number of photons and eventually reaches saturation. The saturation is observed when the number of photons striking the EC-deposited surface is >7.5 × 10^17^ for both the FAPbBr_3_ and the CN surfaces. Cross-section values for the formation of each of the photoproducts were obtained using linear fits of the initial formation rates. For *m*/*z* = 43, one obtains σ = 2.4 ± 0.1 × 10^–19^ cm^2^ on top of the FAPbBr_3_ substrate and σ = 1.0 ± 0.7 × 10^–19^ cm^2^ on top of CN. The saturation of photodecomposition can be explained by surface poisoning induced by the strong adsorption of chlorine atoms following dissociation of EC.^18^ From the linear fitting of the initial growth of the photoproduct, the cross-sectional values for ethane, propyl, and allyl radical formation are (indirectly derived) from Figures S7 and S8 in Supporting Information, as demonstrated in Figure 3 and their numerical values are summarized in Table 1. Practically identical cross-sections were obtained for the formation of the allyl and the propyl, indicating that both are fragmentation radicals that were formed within the QMS ionizer and originate from the same precursor molecule, butane (C_4_H_6_), assumed to be formed on the surface due to the thermal recombination of two partially dissociated ethyl radicals (C_2_H_3_) that were formed upon the EC photodissociation.

**Table 1:** Formation Cross-Sections for Photoproducts (Ethane, Allyl Radical, and Propyl Radical) Following Photon Irradiation at 532 nm of EC, Adsorbed on Top of the Indicated Surfaces^a^

	cross-section values (σ)		
substrate	ethane (mass 30) (mass 30, C_2_H_6_), cm^2^	allyl radical (mass 41, C_3_H_5_), cm^2^	propyl radical (mass 43, C_3_H_7_), cm^2^
FAPbBr_3_	3.6 ± 0.1 × 10^–19^	1.8 ± 0.1 × 10^–19^	2.4 ± 0.1 × 10^–19^
carbon nitride	1.3 ± 0.1 × 10^–19^	1.0 ± 0.1 × 10^–19^	1.0 ± 0.1 × 10^–19^
CsPbBr_3_	6.9 ± 0.1 × 10^–19^	1.7 ± 0.3 × 10^–19^	1.8 ± 0.1 × 10^–19^
MAPbBr_3_	6.0 ± 0.1 × 10^–20^	1.8 ± 0.1 × 10^–20^	2.0 ± 0.4 × 10^–20^

a The results obtained on CsPbBr_3_ and MAPbBr_3_ were taken from ref (18).

#### 3.2.2 Mechanism of EC-Photoinduced Reactivity on FAPbBr_3_ and CN Substrates

The initial stage following the dissociative electron attachment (DEA) process includes photofragmentation of the parent EC molecule to the alkyl radical, C_2_H_5_ (mass 29), and to chloride anion.^18,27,28^ This fragmentation leads to the chemistry and new products we observed as a result of subsequent heating during TPD. The absence of any oxygen-containing products suggests that unlike the methyl chloride case reported previously,^27^ the slightly longer hydrocarbon chain leads to preferred interalkyl chain interactions that result in the formation of hydrocarbons such as butane (and its fragments C_3_H_5_ (mass 41) and C_3_H_7_ (mass 43)). The overall DEA mechanism follows e^–^ + AB → AB^–^* → A + B^–^, in chlorinated hydrocarbons at low electron energies around 2.2 eV.^28^ The low energy electrons arise from 355 nm photoexcitation of the two substrates. It results in a chlorine anion (atom) strongly attached to the substrate and an ethyl radical that upon annealing can further react with its neighbor radicals to form various hydrocarbon products, as discussed above.

Exponential fits are shown in Figure 3 as a comparison. It was shown, however, that these fits lead to significantly higher cross-sectional values, affected by the surface coverage of the chlorine atoms that function as a poison, as previously demonstrated.^18,19^ From the (linear, initial growth) cross-section values, it is clear that photoreactivity on top of the FAPbBr_3_ surface is a factor of 2–3 higher than on the carbon nitride substrate, which is known as a potential photocatalyst at room temperature for water splitting, for example.^22^

It is important to note that at 532 nm there is absolutely no photodecomposition activity on both SiO_2_/Si(100) and Ag nanoparticles deposited on top. For photoreactivity to occur on these surfaces, excitation by pulsed laser UV light (355 nm) was neseccary.^19^

### 3.3 Photoluminescence

**Figure 4:**
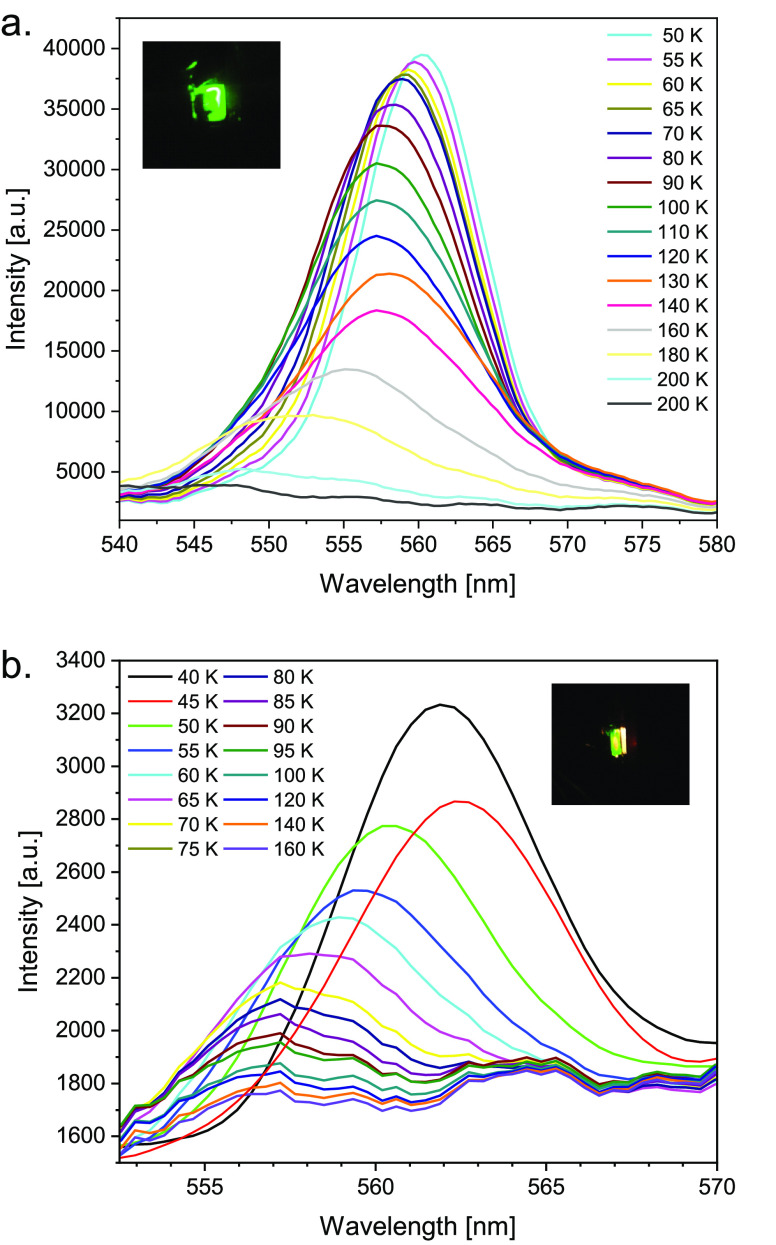
Photoinduced luminescence (PL) of (a) FAPbBr_3_ and (b) carbon nitride (CN) following excitation by 355 nm and laser power of 5 mJ/pulse at the indicated sample temperature between 40 and 160 K. The *y*-axis reflects the actual intensity of the PL light, as detected by an Ocean Optics spectrometer.

In general, photoluminescence (PL) studies may help in the detection of surface states and defects. The recorded PL spectra of the FAPbBr_3_ and carbon nitride films under different substrate temperature conditions are plotted in Figure 4 following excitation by 355 nm photons. The PL peak appears at ∼560 nm for both FAPbBr_3_ and carbon nitride surfaces, demonstrating the temperature-dependent PL spectra of both substrates recorded at temperatures from 40 to 160 K and revealing a blue shift as the temperature increases. In the PL spectra taken from FAPbBr_3_, a single and relatively narrow peak is recorded in most of the temperature range, attributed to the emission from the lowest state exciton only, indicating negligible trap state density.

There is a slight initial blue shift from 560.5 nm (50K) to 558.3 nm (80K), and then the peak position becomes stable at 557.5 nm all the way to 140 K, associated with a decrease in intensity. From 140 to 200 K there is a faster blue shift of the PL peak down to 545 nm where the intensity diminishes.

In contrast, for CN (Figure 4b), 1 order of magnitude lower PL intensity has been recorded with a peak at 562.4 nm, with gradually decreasing intensity as the substrate temperature increases in the range 40–160 K. The peaks at 557.5 and 565.0 nm appear to be stable without further blue shift at temperatures above 80 K but with decreasing intensity as temperature rises. The photochemistry data are found to be consistent with the luminescence results (see below). Higher photoreactivity on top of the FAPbBr_3_ surface relative to the carbon nitride substrate can be attributed to a low density of defects implied from the PL studies.

### 3.4 PL Lifetime Measurements

**Figure 5:**
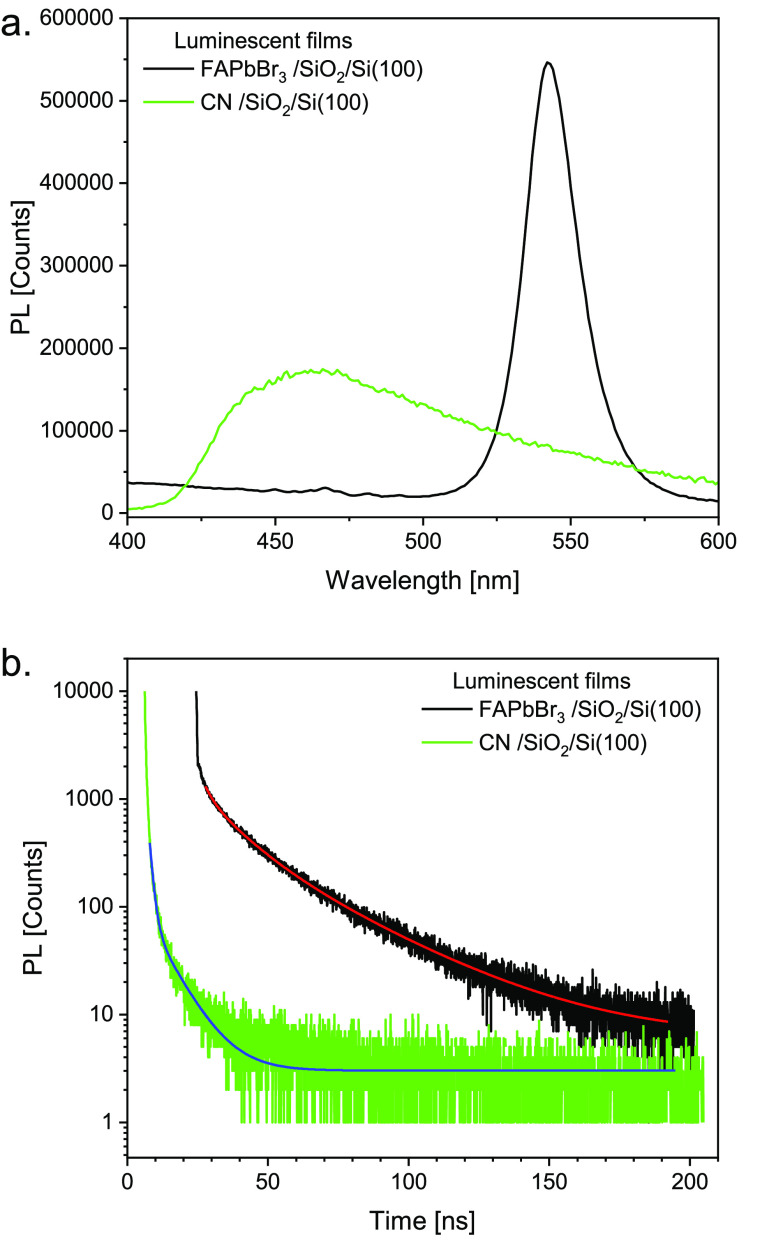
Luminescence (a) and corresponding lifetimes (b) obtained from FAPbB_3_ (black) and CN (green) films deposited on the PSi surface.

Both the FAPbBr_3_ and the carbon nitride films were ex situ excited by a 0.5 ns pulse duration LED at 375 nm excitation wavelength to determine their PL lifetime. The luminescence of the CN sample peaks around 460 nm and its spectral range is relatively wide. That of the FAPbBr_3_ sample is narrower and is peaked at 550 nm. Both PL spectra are demonstrated in Figure 5a. As shown in Figure 5b, the best fit analysis for the FAPbBr_3_ luminescence decay (black curve) is obtained by three different exponents, characterized by lifetimes of 2.5 ± 0.1 ns, 11.3 ± 0.6 ns, and 31.0 ± 1.0 ns. Their relative contributions to the total area under the lifetime peak are 0.29, 0.63, and 0.08, respectively. Similar analysis in the case of the luminescence obtained from the CN film leads to two lifetimes of 1.2 ± 0.1 ns and 8.6 ± 0.2 ns, with relative contributions of 0.8 and 0.2 for the short and the long lifetimes, respectively. A question may arise: what are the dominant and most important surface sites with respect to their role in enhancing the photochemical process? We attribute the more effective photochemistry to sites with longer lifetimes. This assumption is based on a hypothesis in which the excited electrons are dominant in running the electron-induced reactivity via a dissociative electron attachment mechanism. In such a mechanism, the photoreactivity would dominate over the longer excited state lifetime sites vs competing channels of luminescence. The luminescing channel originates from electron–hole recombination back to the ground state. Another open question regarding PL lifetimes is the possible difference between ex situ, ambient lifetimes measurements, as performed here, and measurements conducted under UHV conditions that we could not perform. It is reasonable to assume that in a clean environment, under UHV, longer lifetimes would be obtained.

### 3.5 Comparison of the Photoreactivity Cross-Sections with Previous Work

In Table 1, the photoproducts’ formation cross-sections on top of FAPbBr_3_ and carbon nitride substrates are compared with our previous work, where similar studies were performed on other halide perovskite substrates: methylamine lead bromide (MAPbBr_3_) and cesium lead bromide (CsPbBr_3_).^18^ We have reported the formation cross-sections of *m*/*z* = 43 (propyl radical) to be 2.4 ± 0.1 × 10^–19^ cm^2^ and 1.0 ± 0.1 × 10^–19^ cm^2^ on top of FA and CN substrates, respectively, which is rather similar to our previous study. The formation cross-sections on top of the carbon nitride substrate are generally smaller than all the halide perovskite substrates for all the detected photoproducts. The formation cross-section values for allyl (*m*/*z* = 41) and propyl (*m*/*z* = 43) follow the trend FAPbBr_3_ > CsPbBr_3_ > MAPbBr_3_ > carbon nitride. This trend seems to coincide with the PL intensity: The substrate with stronger PL correlates with a higher photodecomposition cross-section of the probe EC molecules that lead to the butane products (represented by its fragments at *m*/*z* = 41 and *m*/*z* = 43). In contrast, the ethane formation cross-section (at *m*/*z* = 30) is largest on top of the CsPbBr_3_ substrate.

## 4 Conclusions

The main goal of this investigation has been to focus on the role of (very different) substrates on their reactivity toward photodecomposition of a common probe molecule, ethyl chloride. In addition to their rather different chemical structure, their excited-state lifetime, which should directly affect the photochemical activity, is also different: FAPbBr_3_ (τ ∼ 2–30 ns, measured here as a solid film) and CN (τ ∼ 2–9 ns, also as a solid film). While the halide perovskites are known for their excited state transformation into a photovoltaic channel, the CN substrates are known as efficient photocatalysts, e.g., for water splitting, and both are active in the visible spectral range.

Specifically, we have studied the ethyl chloride (EC) photochemistry under UHV conditions, on top of FAPbBr_3_ and carbon nitride films deposited on SiO_2_/Si(100). We found that both substrates enhance the decomposition of ethyl chloride when irradiated by pulsed 532 nm laser excitation, while on the bare SiO_2_/Si(100) substrate no photoreactivity of the EC molecule is recorded. The FAPbBr_3_ halide perovskite substrate has shown the most efficient reactivity with respect to *m*/*z* = 41 and *m*/*z* = 43 fragment formation (both reflect butane parent molecule formation) when compared to CN and two other (CsPbBr_3_ and CH_3_NH_3_PbBr_3_) halide perovskite substrates that were reported in a previous study.^18^ Interestingly, the cross-section for ethane formation (*m*/*z* = 30) was highest on CsPbBr_3_, almost a factor of 2 higher than on FAPbBr_3_. The expected correlation between charge carriers’ recombination lifetime and the photoreactivity yield only partially holds. Other parameters that affect the photofragments’ reactivity and recombination rates on the surfaces of the halide perovskites and CN (e.g., surface defects types and density) apparently also influence the final yield as determined here via postirradiation TPD measurements and overall cross-section determination. The observations reported here provide the basis to explore their use for other photocatalytic applications such as water splitting under the condition that these halide perovskites are protected against damage and deactivation due to water and oxygen.

## Supporting Information Available

Characterization of the FA and CN films (XRD and SEM), TPD spectra of photofragments, cross-section of photofragment formation, FTIR and NMR spectra of the CN films, and TPD of photoproducts following various laser excitation wavelengths (PDF)
