# Supplementary material for: Formamidinium Halide Perovskite and Carbon Nitride Thin Films Enhance Photoreactivity under Visible Light Excitation
Source: J Phys Chem A. 2022 Jun 2;126(23):3724–31. doi: 10.1021/acs.jpca.2c02565 (PMC9207933; doi:10.1021/acs.jpca.2c02565)
Supplement: Supplementary file 1 — jp2c02565_si_001.pdf [file jp2c02565_si_001.pdf]

Formamidinium Halide-Perovskite and Carbon Nitride Thin Films Enhance Photo-reactivity Under Visible Light Excitation

Gopi Ragupathy[1], Julian Rieß[2], Bat-El Cohen[1], Lioz Etgar[1], Roey Sagi[1],

Kumar P. Deepak[1], Reinhard Schomäcker[2] and Micha Asscher[1]*

[1]Institute of Chemistry, Edmund J. Safra Campus, Givat-Ram, The Hebrew University of Jerusalem, 91904 Israel

[2] Department of Multiphase Reaction Technology Technical Chemistry, Institute for Chemistry of the TU Berlin

* Corresponding author e-mail: micha.asscher@mail.huji.ac.il

KEYWORDS: Visible Light Surface-Photochemistry, Carbon nitride, Perovskite surfaces, Photoluminescence, Luminescence lifetime

Supporting Information:

Formamidinium lead bromide perovskite ($FAPbBr_3$) thin film was deposited on a porous silicon substrate for enhanced adhesion. The halide perovskite was prepared by one sequence deposition technique, using the solvent engineering method as described below, where X-ray diffraction (XRD) confirms crystallinity as a result of the single phase formation of $FAPbBr_3$ thin film. Furthermore, the typical absorption curve of $FAPbBr_3$ halide perovskite structure is presented as well. The halide perovskite deposition method was optimized to obtain closely packed, smooth and uniform film on top of a glass substrate. Subsequently, this process was applied on a pours silicon substrate, which was then used for the photochemistry experiments presented in this work within ultra-high vacuum eneivronment.

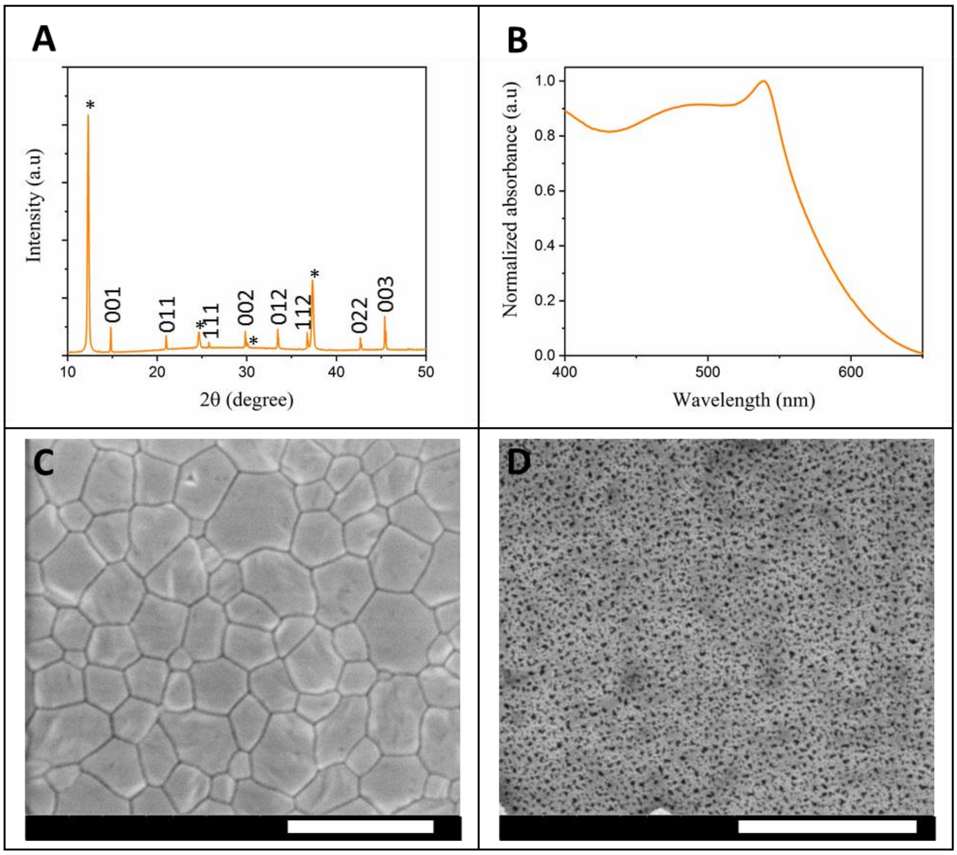

Figure S1: (A) $FAPbBr_3$ perovskite XRD diffraction (marked in star is the $PbBr_2$ residues), and (B) absorbance curve. (C) Top-view SEM image of $FAPbBr_3$ thin film on glass substrate (scale bar 1 µm) and (D) porous silicon substrate (scale bar 400nm).

1. Experimental methods

1.1 Halide perovskite film deposition:

FAPbBr3 perovskite film was prepared in a nitrogen flow-glove box by using solvent engineering technique reported elsewhere, e.g. main text Ref. 5. 50µL of the filtered perovskite solution (PTFE 45um) spin-coated on porous Si film using dynamic spin program: 5 sec loading time followed by 10sec spin at 1000 rpm, which increase to 5000rpm for 50 sec. 30 sec before the program, ended, 100 µL of anhydrous chlorobenzene (Aldrich) was dropwise on the substrate. For complete crystallization,

the film was annealed 30 minutes at 100°C. Perovskite solution contains 1M of $PbBr_2$ (Aldrich) and FABr (1:1) in DMF: DMSO solvents (85:15).

1.2 X-ray diffraction (XRD):

XRD measurements were performed on the D8 Advance diffractometer (Bruker AXS, Karlsruhe, Germany) with a secondary graphite monochromator, 2° Soller slits, and a 0.2 mm receiving slit. XRD patterns within the range 2°–75° 2q were recorded at room temperature using CuKa radiation (λ = 1.5418 Å) with the following measurement conditions: tube voltage of 40 kV, tube current of 40 mA, step-scan mode with a step size of 0.02° 2θ and counting time of 1 s per step.

1.3 (XHRSEM):

SEM images obtained by using Magellan XHRSEM was performed using an FEI (Field mission Instruments), The Netherlands.

1.4 Absorption Measurements:

Absorption measurements were performed using Jasco V-670 spectrophotometer.

2. Carbon nitride synthesis:

2.1 XRD:

Powder XRD measurements of CN films (See Figure S2) were performed on a Bruker AXS D8 advanced diffractometer equipped with a position-sensitive detector (PSD), curved germanium (111) primary monochromator, and a Cu-Kα (λ = 1.5418 Å) radiation source. The XRD patterns indicate one main peak at 2ϴ = 27.4° which is ascribed to the distance between the layers of the material and a smaller peak at 2ϴ = 13.1° which indicates interplanar repetition of tri-s-triazine units.

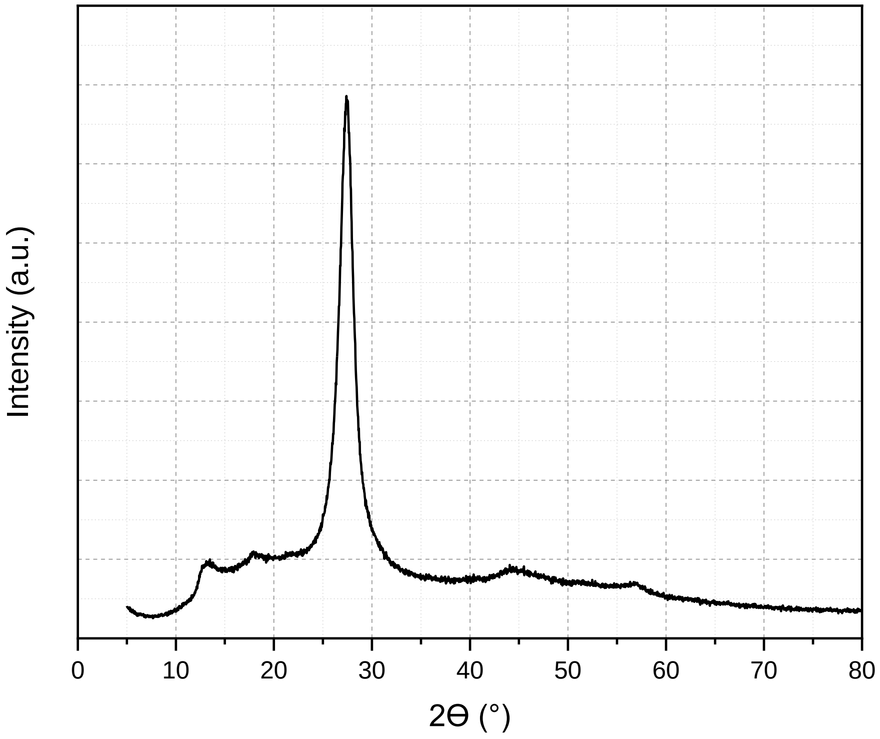

Figure S2: X-ray powder diffraction patterns of carbon nitride. Explanation and assignment in the text abovs.

2.2 Scanning electron microscopy (SEM):

SEM images of CN films (Figure S3) were obtained using a JEOL JSM-7401F high-resolution field emission SEM operated at 10 kV.

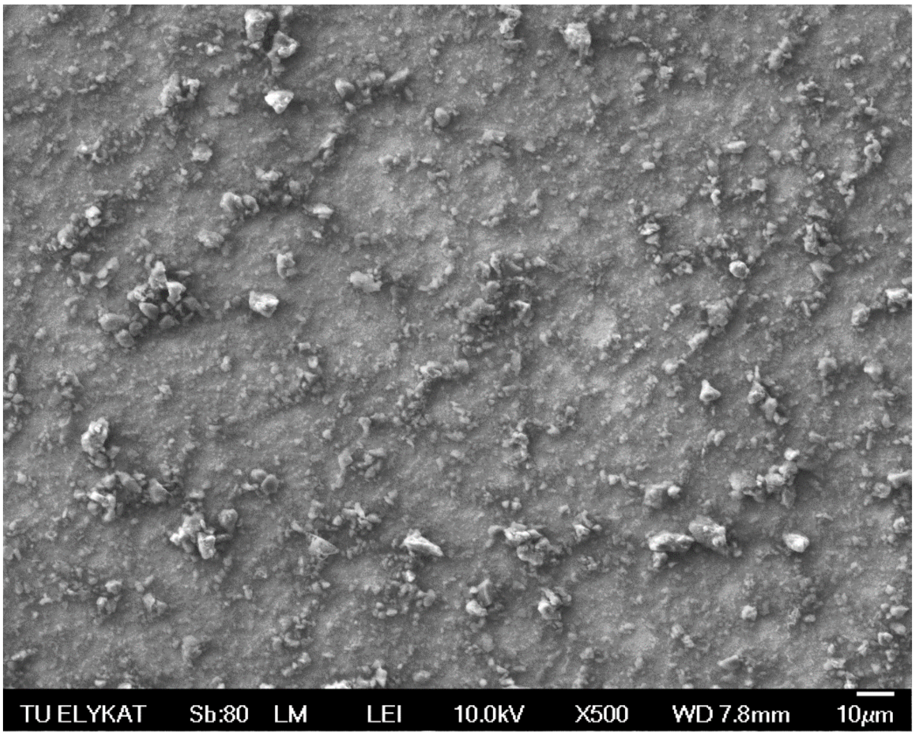

Figure S3: Scanning electron microscopy image of carbon nitride, indicating the level of uniformity of the CN films.

2.3 UV-Vis:

Diffuse reflectance UV-Vis absorption spectra of the CN samples were measured using a LAMBDA 650 UV/Vis spectrophotometer with a 150 mm integrating sphere (Perkin Elmer, USA). The spectrum (Figure S4) reveals a strong absorption band in the violet blueish region at an onset of 450 nm, with two maxima at 350 nm and 250 nm. This band corresponds to the bandgap transition from N2p to C2p orbitals. Note the slight difference compared to the lower resolution obtained from the lifetime measurement instrument described in the main text, Figure 5. There the absorption spectrum was taken from a thin film (and different instrument), therefore the separation between the two peaks revealed in Figure S4 is not resolved.

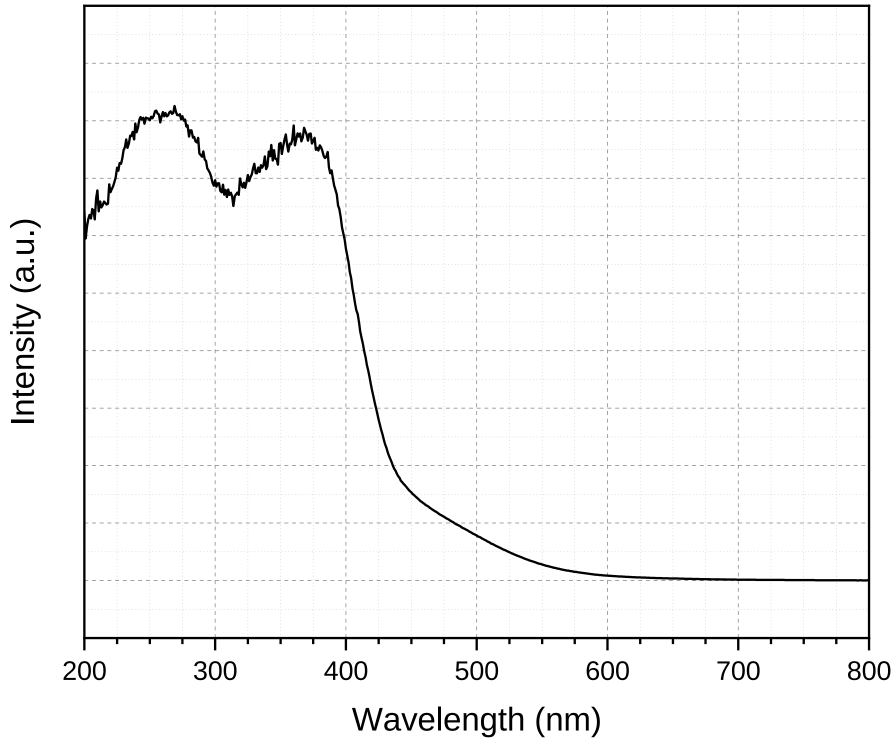

Figure S4: Diffuse reflectance UV-Vis spectra of carbon nitride (CN), applying an integrated sphere capability.

2.4 ^{13}C solid-state NMR:

Solid-state $^{13}C\{^{1}H\}$ cross-polarization magic angle spinning (CP/MAS) NMR measurements were performed by using a Bruker Advance 400 spectrometer. The MAS frequency was 10 kHz, the ^{13}C was proton-decoupled using two-pulse phase-modulated (TPPM) decoupling and the contact time for the ^{13}C CP measurement was 2 ms. In Figure S5 the recorded spectrum shows two main signals at around 154 ppm and 163 ppm indicating the formation of heptazine (Tri-s-triazine) units.

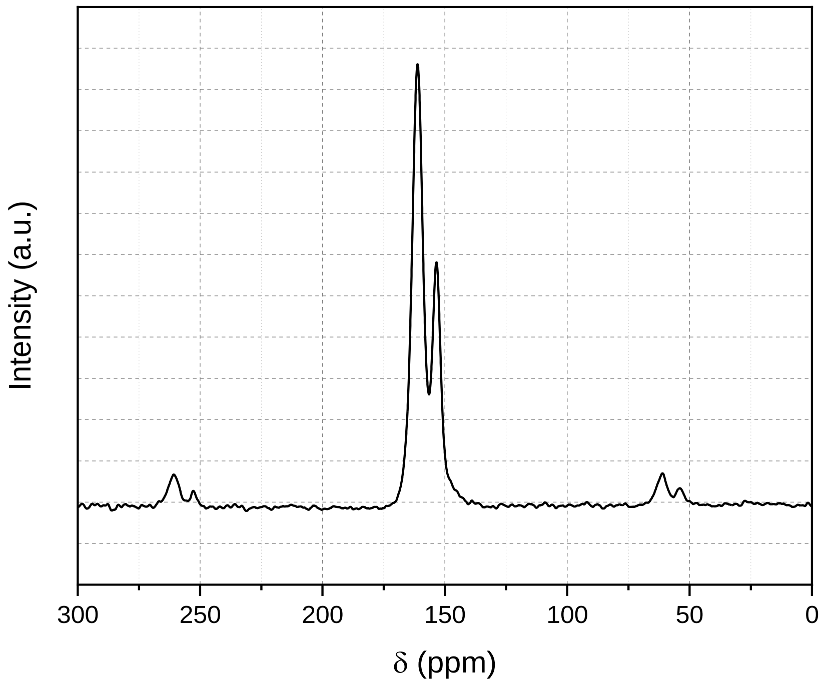

Figure S5: ^{13}C solid-state NMR of carbon nitride. Assignment of the two peaks is discussed above.

2.5 Fourier transform infrared (FTIR):

FTIR spectroscopy of the carbon nitride sample was performed with a Varian 640IR spectrometer equipped with an attenuated total reflection (ATR) cell.

The FTIR spectra (Fig. S6) exhibits all characteristic stretching modes of aromatic CN heterocycles (e.g. C-N(-C)-C and C-NH-C) at 1200 cm^{-1} – 1600 cm^{-1}. In addition, the breathing modes of heptazine units are visible at 810 cm^{-1}. Next, a small peak at ~2350 cm^{-1} arises which can be attributed to adsorbed CO_2 from the atmosphere [K. L. Kauffman, J. T. Culp, A. Goodman and C. Matranga, *J. Phys. Chem. C*, **2011**, 115, 1857]. The broad peaks between 3000 cm^{-1} and 3600 cm^{-1} arise from N-H stretching which confirms the existence of NH_2 groups at the surface.

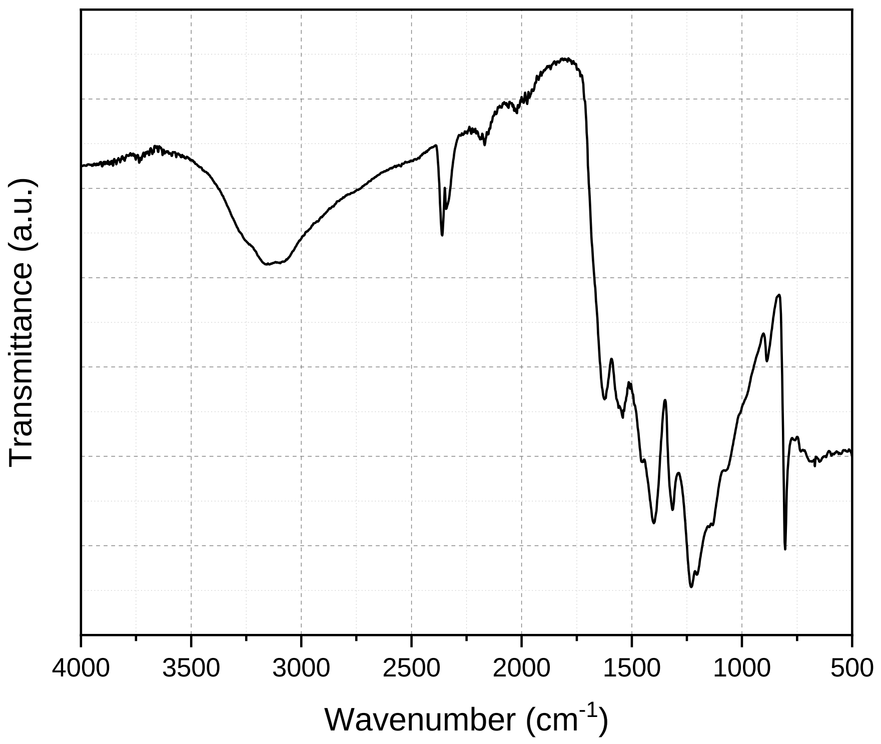

Figure S6: Fourier transform infrared spectra of carbon nitride. Assignments of the IR peaks is discussed above the figure.

2.6 Photo-chemistry of adsorbed EC molecules employing visible-light (532 nm):

Integrated area under TPD peaks of EC (mass 30, 41, 43) from $FAPbBr_3$ /SiO_2/Si(100) and CN/SiO_2/Si(100) samples are shown in Figures S7 and S8.

Temperature dependence of the electron excited luminescence property of $FAPbBr_3$ (not shown) was observed to be spectrally almost identical to that of the photoluminescence, as previously reported (see reference 18 in the main text). However, in addition to the counterintuitive blue shift and signal quenching with increasing temperature, the electron-induced luminescence intensity is found to be about five times higher than the photon excitation. Moreover, It should be noted that

the rate of electrons ($1\mu A = 6.3\times10^{12}$ electron/sec) striking the $FAPbBr_3$ substrate for electron induced luminescence is four order of magnitude smaller than that of photons/sec (number of photons/sec for 355 nm laser was 1.79×10^{16} /sec) for the photon induced luminescence. Hence the general cross section for electron induced luminescence is several orders of magnitude larger than that of photo-induced luminescence. At the same time the signal quenches drastically with increasing temperature, compared to that of photon excited luminescence. Almost half of the signal was diminished when the temperature was increased from 40 to 50 K and it completely vanished above 150 K. This luminescence quenching can be attributed to the dominance of the electron-phonon coupling over thermal expansion. There is no evidence for the presence of any defects or phase transition. Another interesting observation was the electron energy dependence (not shown) where the luminescence intensity rapidly quenched as electron energy decreased, with total quenching below 500 eV electrons energy (see reference 18 in the main text). This observation is not clear at the moment and requires further study.

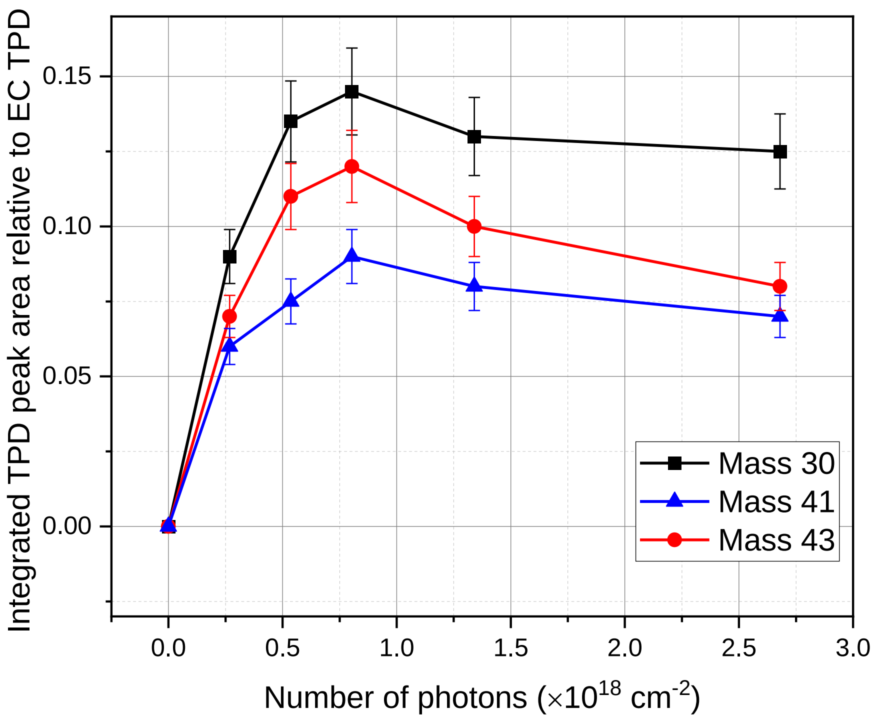

Figure S7. Integrated TPD peaks of Ethane, mass 30 (C_2H_6), Allyl radical, mass-41 (C_3H_5, a fragment of Butane) and Propyl radical, mass-43 (C_3H_7, a fragment of Butane) vs. the number of visible (532 nm) light excitation photons striking the halide perovskite, $FAPbBr_3$ samples

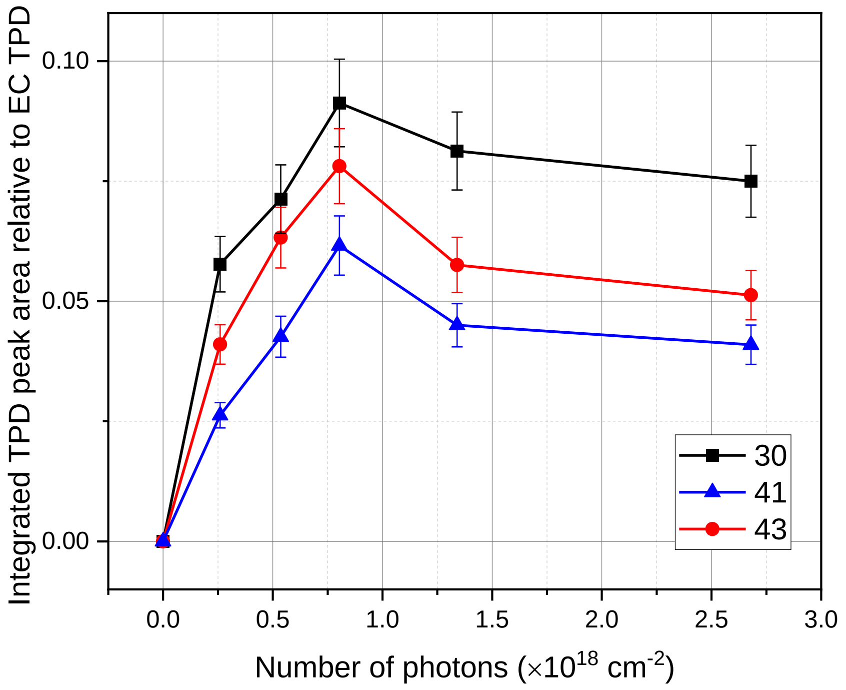

Figure S8. Integrated TPD peaks of Ethane, mass 30 (C_2H_6), Allyl radical, mass-41 (C_3H_5, a fragment of Butane) and Propyl radical, mass-43 (C_3H_7, a fragment of Butane) vs. the number of visible (532 nm) light excitation photons striking the carbon nitride (CN) samples.

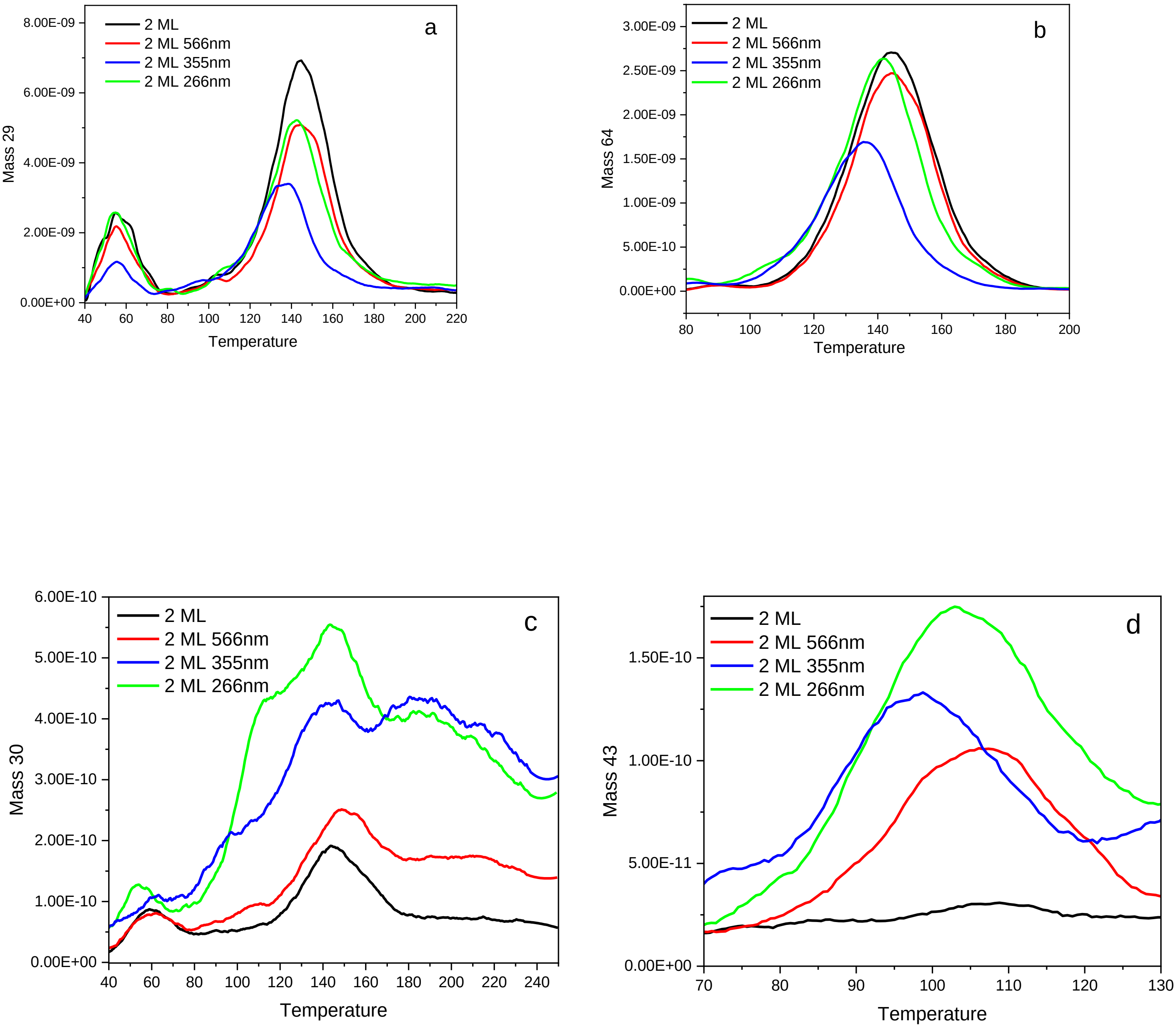

Figure S9. Post irradiation TPD spectra of **: parent molecule** (mass 29 – ethyl, electron impact fragment of the parent ethyl chloride (EC) molecule; mass 64, the parent C_2H_5Cl (EC) molecule and **photo-products**: (mass 30, C_2H_6 (ethane) and mass 43, C_3H_7 – Butyle, an electron impact fragment of Butane). All spectra are shown for the indicated excitation wavelengths (266 nm, 355 nm, 532 nm) and the same laser power with EC on CN substrate. The heating rate was 2 K/sec.
